# 

**Published:** 1867-06

**Authors:** 


### CONTENTS.

### ORIGINAL COMMUNICATIONS.

Do Gum Plates injure our Taste.... 235
Absolutely pure Gold for filling Teeth.... 242
President's Address to the Mississippi Valley Association, March 6, 1867.... 244
Root of Tooth in the Antrum of Highmore.... 248
Cracking of Rubber Plates.... 249

### PROCEEDINGS OF SOCIETIES.

Extract from Proceedings of the Mississippi Valley Dental Association.... 251
Extracts from Discussions of the first meeting of the Ohio State Dental Society.... 253
Indiana State Dental Association.... 262
Old Colony Dental Association.... 264
Hudson River Association of Dental Surgeons.... 265
Ohio State Dental Society.... 266
Delaware Dental Association.... 266
Chicago Dental Society.... 267
The Iowa State Dental Society.... 267

### EDITORIAL.

Teeth Extracted without Pain.... 269
The Code of Ethics.... 271
Death of Dr. John McCurdy.... 273
The American Journal of Dental Science.... 274
American Dental Association.... 274
Ohio State Dental Society.... 275
Specialties in Medicine.... 275
Convention of Medical Teachers.... 278
The American Medical Association.... 279
American Dental Association.... 280

